# Highly Efficient Isolation of *Populus* Mesophyll Protoplasts and Its Application in Transient Expression Assays

**DOI:** 10.1371/journal.pone.0044908

**Published:** 2012-09-13

**Authors:** Jianjun Guo, Jennifer L. Morrell-Falvey, Jessy L. Labbé, Wellington Muchero, Udaya C. Kalluri, Gerald A. Tuskan, Jin-Gui Chen

**Affiliations:** Biosciences Division, Oak Ridge National Laboratory, Oak Ridge, Tennessee, United States of America; University College Dublin, Ireland

## Abstract

**Background:**

*Populus* is a model woody plant and a promising feedstock for lignocellulosic biofuel production. However, its lengthy life cycle impedes rapid characterization of gene function.

**Methodology/Principal Findings:**

We optimized a *Populus* leaf mesophyll protoplast isolation protocol and established a *Populus* protoplast transient expression system. We demonstrated that *Populus* protoplasts are able to respond to hormonal stimuli and that a series of organelle markers are correctly localized in the *Populus* protoplasts. Furthermore, we showed that the *Populus* protoplast transient expression system is suitable for studying protein-protein interaction, gene activation, and cellular signaling events.

**Conclusions/Significance:**

This study established a method for efficient isolation of protoplasts from *Populus* leaf and demonstrated the efficacy of using *Populus* protoplast transient expression assays as an *in vivo* system to characterize genes and pathways.

## Introduction

The establishment of renewable energy sources is essential for energy security and reduction of CO_2_ emissions. Among all of the options, production of lignocellulosic ethanol from plant feedstock has become one of the favorable approaches [1], [2], [3], [4]. *Populus* is characterized by a rapid growth rate, high productivity and wide adaption to environment and therefore, has become one of the promising bioenergy feedstocks [1], [5], [6]. With the availability of its full genome sequence [7] and the establishment of transgenic techniques [8], [9], [10], *Populus* is also emerging as a model organism for studying woody plants [11].

One of the rate-limiting steps in studying the function of genes in *Populus* is its long life cycle (e.g., it takes a minimum 6 to 12 years to reach reproductive maturity [7]). Furthermore, it typically takes four to eight months from inoculation to generate rooted seedlings via *Agrobacterium*-mediated transformation approach [12]. Therefore, the capacity of conventional genetic manipulation in *Populus*, one of the most commonly used approaches for studying gene function, is largely limited by the lengthy time required to obtain suitable genetic materials. This limitation is more pronounced when dealing with cellular signaling events that often involve multiple gene products.

Leaf mesophyll protoplasts have been developed as an informative system in Arabidopsis and a few other plant species for rapid dissection of signaling events that lead to the regulation of gene expression, as well as for many other molecular characterizations, such as protein-protein interaction, subcellular localization, and protein-DNA interaction [13], [14]. Arabidopsis leaf mesophyll protoplasts have also been used to study the function of *Populus* genes [15], [16]. However, the inherently different genomic, physiological and developmental characteristics between a herbaceous plant and a perennial tree species may render Arabidopsis protoplasts a less ideal system for dissecting the signaling events that regulate the development of *Populus*.

In this paper, we described the micro-propagated *Populus* grown on MS media as an excellent alternative starting material for the efficient isolation of leaf mesophyll protoplasts. We also optimized enzyme concentrations and digestion time for efficient protoplast isolation. We then established an efficient *Populus* protoplast transient expression system and demonstrated the efficacy of using this *in vivo* system to study the activity of *Populus* genes.

## Results and Discussion

### Isolation of Protoplasts from *Populus* Leaves

**Figure 1:**
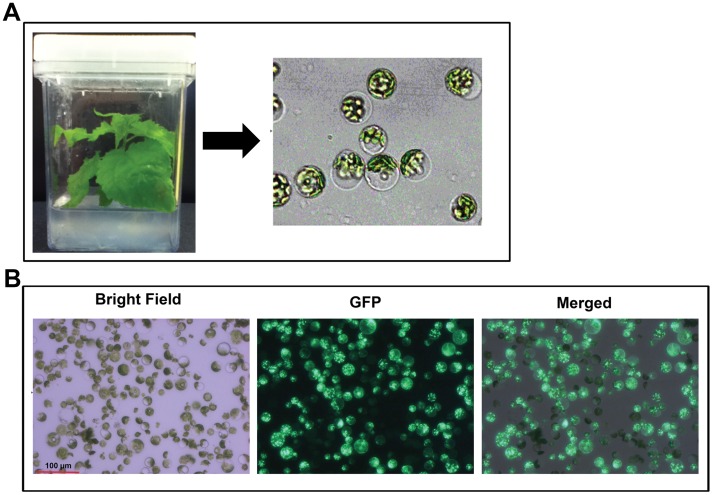
*Populus* leaf mesophyll protoplasts. (**A**) Optimal yield and quality of protoplasts can be isolated from one month-old *Populus* plants grown on MS medium in a magenta box. (**B**) High transfection efficiency is indicated with GFP signal.

A protocol for the isolation of Arabidopsis mesophyll protoplasts has been well established [14], [17], [18]. There have been three reports on isolation of protoplasts from *Populus*. Two of them reported the isolation of protoplasts from *Populus* cell suspension culture [19] or leaves [20], and the other used *Populus* protoplasts to study protein subcellular localization of *Populus* salt overly sensitive genes [21]. In the latter study, it was briefly mentioned that an essentially same procedure was used as that in Arabidopsis [21]. Because this is no *Populus* cell suspension culture available, we tried to establish a robust protocol for isolating protoplasts from *Populus* leaves. Initially, we tried to isolate protoplasts from leaves of soil-grown *Populus* plants cultivated in a greenhouse. Newly emerged leaves from two different *Populus* clones (*Populus trichocarpa* clone Nisqually-1 and *P. tremula* X *alba* clone 717-1B4) were used for the study. No or very few protoplasts could be isolated from these plants (data not shown). Field or greenhouse-grown *Populus* under adequate irradiation conditions usually produce leaves with a thick cuticle and recalcitrant cell walls, which are likely to be the major factors limiting protoplast isolation. Because cuticle thickness is known to increase with high light intensity and low relative humidity [22], we then propagated *Populus* 717 on MS medium in a Magenta box through shoot propagation under relatively low light (70 µmol m^−2 ^s^−1^) conditions [23]. The propagated *Populus* plants were able to produce fully expanded and healthy-looking leaves (Figure 1A). We found that protoplasts with high yield and quality could be isolated from these leaves (Figure 1). Evans blue staining indicated that more than 90% of the protoplasts isolated were viable (n>500) (Figure S1).

More importantly, when we followed the same protocol for isolating protoplasts from leaf mesophyll cells of *Populus* plants grown in a greenhouse or a Magenta box as that used in Arabidopsis, we found that the yields and quality of protoplasts were very poor. Therefore, we modified the protocol by adjusting enzyme concentrations and digestion time. We found that twice the enzyme concentration and a longer digestion time (4–5 h) are required for an optimal yield of protoplasts from *Populus* leaves. We could routinely obtain 1×10^7^ protoplasts from one-gram (fresh weight) leaf tissue in 10 ml of enzyme solution. The high yield of protoplasts permits the application of this system for transfection studies [14].

### Establishment of a *Populus* Protoplast Transient Expression System

We next examined whether *Populus* protoplasts can be efficiently transfected. The protoplasts were transfected with a 35S::GFP (green fluorescent protein) construct using a PEG (Polyethylene glycerol)-mediated transfection approach [14]. In general, a greater than 50% transfection efficiency is required for obtaining reliable and reproducible results with the protoplast system [14]. After incubation overnight, a strong GFP signal could be observed in more than 70% (n>1,000) of the protoplasts transfected with a 35S::GFP construct (Figure 1B). These results indicated that the transfection efficiency of *Populus* protoplasts was sufficiently high to be used for molecular assays.

### Transiently Expressed Organelle Markers are Correctly Localized in the *Populus* Protoplasts

**Figure 2:**
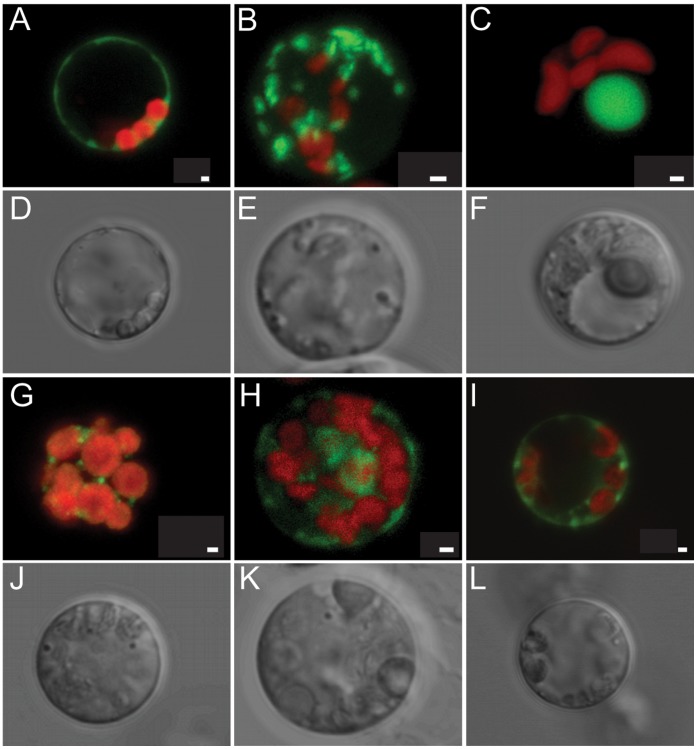
Subcellular localization of various organelle markers in *Populus* protoplasts. (**A**) Plasma membrane; (**B**) Golgi apparatus; (**C**) Nucleus; (**G**) Peroxisome; (**H**) endoplasmic reticulum (ER); (**I**) An ubiquitously-localized protein (RACK1, Receptor for Activated C-protein Kinase 1). Shown in (**D**), (**E**), (**F**), (**J**), (**K**) and (**L**) are bright field images for fluorescent images of (**A**), (**B**), (**C**), (**G**), (**H**) and (**I**), respectively. The organelle markers were fused with mCherry fluorescent protein, and RACK1 was fused with YFP fluorescent protein. The mCherry signal was separated from chloroplast autofluorescence using spectral imaging and linear unmixing. The mCherry and YFP signals are false-colored green and the chloroplast autofluorescence is shown in red. Scale bar, 1 µm.

The next question we asked was whether the products of exogenously-introduced genes can be targeted to the proper subcellular locations. To test this, we expressed several well-known organelle-targeting proteins in *Populus* protoplasts [24] including a plasma membrane marker (Figure 2A), a Golgi apparatus marker (Figure 2B), a nucleus marker (Figure 2C), a peroxisome marker (Figure 2G), an ER (endoplasmic reticulum) marker (Figure 2H), and an ubiquitously-localized protein (RACK1, Receptor for Activated C-protein Kinase 1) (Figure 2I) [25]. After overnight incubation for protein expression, all of the proteins were correctly targeted (Figure 2). These results indicate that the *Populus* protoplast system is suitable for studying protein subcellular localization as well as function.

### Use the *Populus* Protoplast Transient Expression System to Study Protein-Protein Interaction

**Figure 3:**
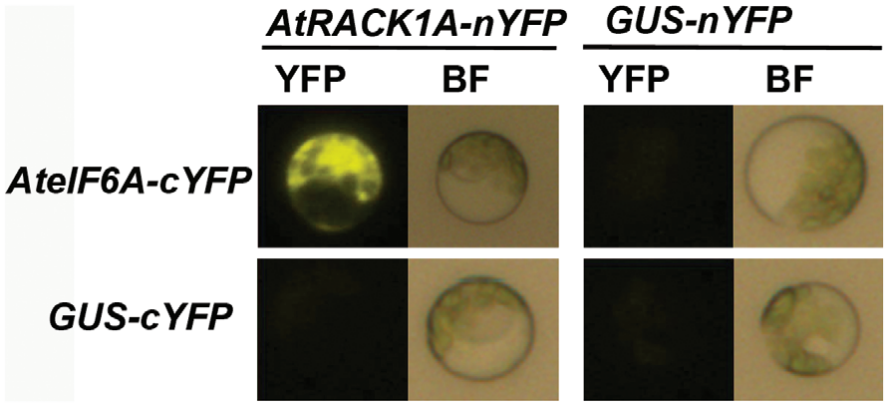
Using *Populus* protoplast system to test protein-protein interaction. Arabidopsis RACK1A (AtRACK1A) and eIF6A (AteIF6A) were fused with the N-terminal and C-terminal half of YFP, respectively. GUS-nYFP/cYFP fusions were used as negative controls. YFP, yellow fluorescent protein. BF, Bright field.

In order to examine whether the *Populus* protoplast transient expression system can be exploited to study protein-protein interaction, a previously identified interaction between *Arabidopsis* RACK1A and eIF6A (eukaryotic initiation factor 6A) [25] was tested in *Populus* protoplasts using a bi-molecular fluorescence complementation (BiFC) approach [26]. AtRACK1A was fused with the N-terminal half of YFP (Yellow Fluorescent Protein) and AteIF6A was fused with the C-terminal half of YFP. When both constructs were co-transfected into *Populus* protoplasts, a yellow fluorescent signal was detected (Figure 3), indicating a positive interaction between these two proteins. As a negative control, GUS (β-glucuronidase) was not found to interact with either RACK1A or eIF6A (Figure 3). These results demonstrated the usefulness of *Populus* protoplast system for studying protein-protein interactions.

### *Populus* Protoplasts are Responsive to Exogenous Plant Hormone Treatments

**Figure 4:**
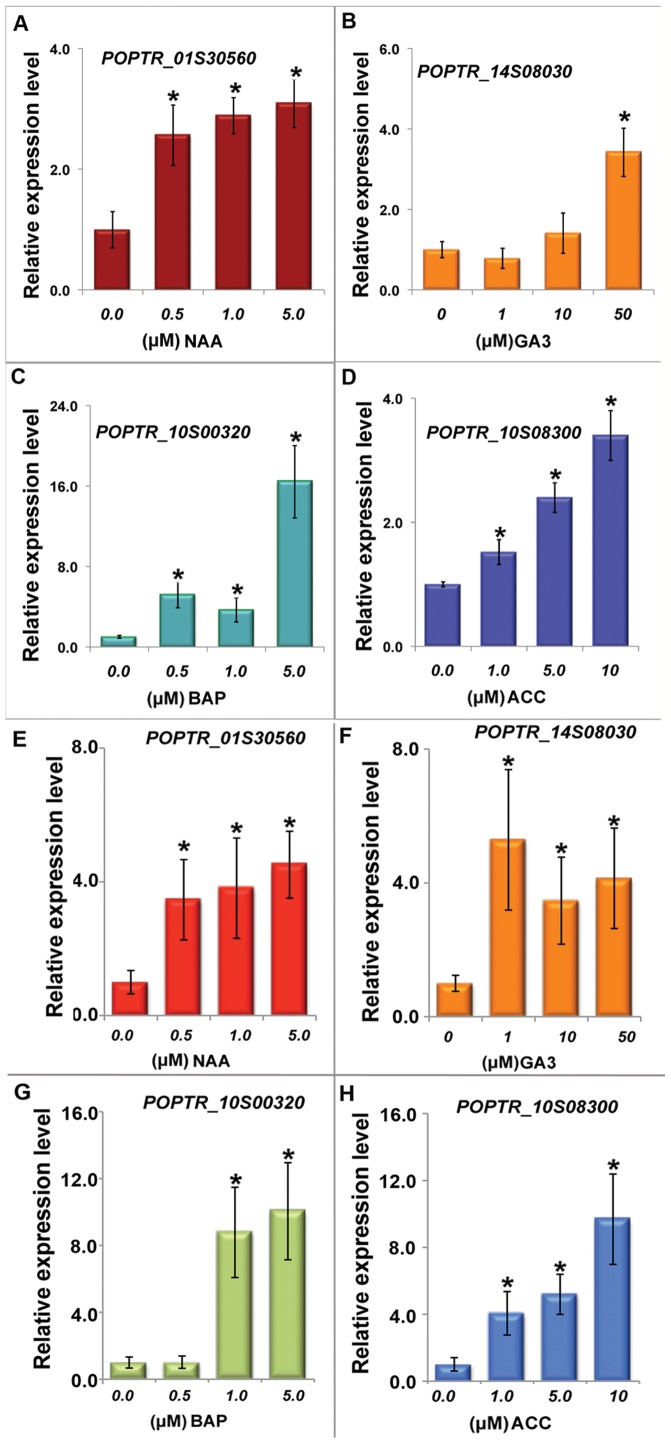
The response of *Populus* protoplasts to various plant hormone treatments. Shown are the change *POPTR_01s30560* transcript in response to different concentrations of NAA in protoplasts (A) and intact leaves (E), the change of *POPTR_14s08030* transcript in response to different concentrations of GA_3_ in protoplasts (B) and intact leaves (F), the change of *POPTR_10s00320* transcript in response to different concentrations of BAP in protoplasts (C) and intact leaves (G), and the change of *POPTR_10s08300* transcript in response to different concentrations of ACC in protoplasts (D) and intact leaves (H). The protoplasts or intact leaves were incubated with various concentrations of plant hormones for 3h before being harvested for qRT-PCR analysis. The experiments were repeated three times with similar results. The averages of three technical replicates ± standard errors are shown. * indicates a significant difference (at P≤0.01, student’s t-test) between each treatment and the untreated control.

It has been shown that plant hormones can trigger the expression of downstream genes of the respective signaling cascades [27], [28], [29], [30] in Arabidopsis mesophyll protoplasts. Thus, the Arabidopsis mesophyll protoplasts have been used as an *in vivo* system for studying the dynamic interaction of molecules in many signaling pathways. We therefore examined the transcriptional response of *Populus* protoplasts to exogenous plant hormone application. We focused on the hormones that were known to play important roles in xylogenesis, including auxin, gibberellin (GA), cytokinin and ethylene [31]. The *Populus* homologues of *AthGH3.2* (*POPTR_01s30560*), a commonly used molecular marker for auxin response, was used as a reporter for auxin response [29]. Its expression was up-regulated 3.5 fold in response to 5 µM NAA (1-naphthaleneacetic acid, a synthetic auxin) treatment (Figure 4A). For gibberellin responses, *POPTR_14s08030* was chosen because it is a close homologue of *At2g45900*, a direct target of the DELLA protein that can be induced by GA treatment in Arabidopsis [32]. An approximately 4-fold increase of its expression was observed in response to 50 µM GA_3_ (gibberellic acid) treatment (Figure 4B). *POPTR_10s00320*, also known as *PtRR13*, is a type B Arabidopsis Response Regulator (ARR) that was reported to respond to the treatment of BAP (6-benzylaminopurine, a synthetic cytokinin) in *Populus* leaves [33]. The expression of *PtRR13* increased 6 to 16 fold in response to 0.5 to 5 µM BAP treatments (Figure 4C). Finally, a *Populus* homologue of Arabidopsis type-B ARR, *POPTR_10s08300*, was chosen as a molecular marker for ethylene response [34]. About a 4-fold increase was observed in protoplasts treated with 10 µM ACC (1-aminocyclopropane-1-carboxylic acid, a precursor for ethylene) (Figure 4D). Auxin [35], ethylene [34], gibberellin [36], and cytokinin [37] have also been reported to promote secondary growth via stimulating cambial cell division in *Populus*. The validation of the presence of these hormone signaling cascades in protoplasts enables us to study their potential roles in the regulation of secondary cell wall biosynthesis in the future.

In order to determine whether the *Populus* protoplasts respond to exogenous hormone treatment in a similar manner as that in intact leaves, we further examined the induction of these genes by plant hormones in intact *Populus* leaves. All these genes responded to the respective hormone treatments as they did in protoplasts (Figure 4E-H). Moreover, the level of transcriptional induction by plant hormones was largely similar between in protoplasts and in intact leaves.

### Use the *Populus* Protoplast Transient Expression System to Study Signaling Events

In order to further examine the feasibility of utilizing *Populus* leaf mesophyll protoplasts to study signal transduction events, we explored an energy-signaling event that was discovered using Arabidopsis mesophyll protoplasts [38]. AthKIN10 (SNF1 kinase homolog 10) is recognized as an energy sensor that perceives the cellular energy status and reprograms the transcriptome for cellular energy homeostasis. One of the reporter genes in Arabidopsis that was up-regulated by AthKIN10 is *DIN6* (*At3g47340*, Dark Inducible 6). We searched the *Populus* genomic information in Phytozome (http://www.phytozome.net/) and found three sequence homologues of DIN6: *POPTR_09s07710.1* (*PtrDIN6-1*, with 91.1% similarity), *POPTR_01s28500.1* (*PtrDIN6-2*, with 91.3% similarity) and *POPTR_05s07720.1* (*PtrDIN6-3*, with 82% similarity). The transcript of *PtrDIN6-3* was not detected in protoplasts under our experimental conditions, and therefore was not studied further.

**Figure 5:**
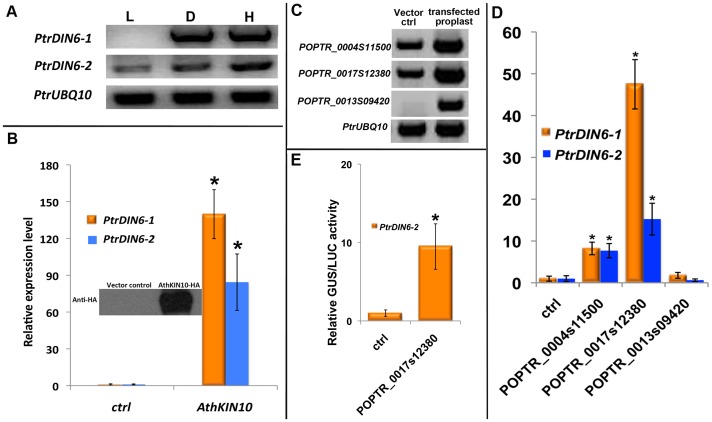
Energy sensing signaling in *Populus* protoplasts. (**A**) Semi-quantitative RT-PCR analysis of *PtrDIN6* transcripts in response to dark and hypoxia treatments. L-Light; D-Dark; H-Hypoxia. The *PtrUBQ10* gene was used as a control. (**B**) The change of *PtrDIN6* transcripts in response to overexpression of AthKIN10 protein. After transfection, protoplasts were incubated overnight to allow the expression of AthKIN10 before samples were harvested for qRT-PCR and western blot analysis. Western blot was used to detect the presence of the introduced HA-tagged AthKIN10 protein. The experiments were repeated three times with similar results. The averages of three technical replicates ± standard errors are presented in the graph. * indicates a significant difference (at P≤0.01, student’s t-test) between protoplasts expressing AthKIN10 and the control (ctrl). (**C**) The expression of three *Populus KIN10* homologues in transfected protoplasts examined by semi-quantitative RT-PCR. The expression of *PtrUBQ10* was used as an internal control. (**D**) The response of *PtrDIN6* transcript to the overexpression of three *Populus KIN10* homologues. The experiments were repeated three times with similar results. The averages of three technical replicates ± standard errors are shown. Protoplasts transfected with an empty vector was used as control (ctrl) for each comparison. (**E**) The activation of *PtrDIN6* by PtrKIN10 in a GUS reporter assay. For each co-transfection, a 35S::LUC (Luciferase) was included and the LUC activity was used to normalize GUS activity to account for the potential variations in the transfection efficiency. The averages of three technical replicates ± standard errors are shown. * indicates a significant difference (at P≤0.01, student’s t-test) between each treatment and the control (ctrl).

We first examined the response of *PtrDIN6-1* and *PtrDIN6-2* to dark and hypoxia, conditions that create cellular energy starvation. We found that both *DIN6* homologues showed high induction by these two conditions (Figure 5A). We then expressed AthKIN10 in *Populus* protoplasts to examine the effect of this energy sensor on the expression of *PtrDIN6* genes. We found that both *PrtDIN6* genes were strongly induced by ectopic expression of AthKIN10 (Figure 5B).

We further extended the experiments to test the effect of transient overexpression of *Populus* homologues of *AtKIN10*. Three *KIN10* homologues were identified via phytozome: *POPTR_04S11500*, *POPTR_17S12380* and *POPTR_13S09420*. While both *POPTR_04S11500* and *POPTR_17S12380* were expressed ubiquitously, *POPTR_13S09420* had extremely low expression (Figure 5C, Figure S2). We found that similar to AtKIN10, overexpression of both *POPTR_04S11500* and *POPTR_17S12380* induced the expression of *PtrDIN6* genes in *Populus* protoplasts (Figure 5D).

To further validate the induction of *PtrDIN6* by *PtrKIN10*, we constructed a reporter cassette in which the genomic DNA sequence that is 2 kb upstream of the start codon of *PtrDIN6*-2 was fused with GUS reporter gene. When both *POPTR_0017s12380* and the *PtrDIN6-2* promoter fused with GUS reporter were co-transfected into the *Populus* protoplasts, we detected a 10-fold increase of GUS activity (Figure 5E), indicating that *Populus* KIN10 protein encoded by *POPTR_0017s12380* can be recruited to the promoter region of *PtrDIN6-2* to activate the expression of *PtrDIN6-2.*

Taken together, these results suggested the presence of a potentially conserved energy sensing signaling cascade mediated by KIN10 in *Populus*, and further demonstrated the suitability of using *Populus* protoplasts to study signal transduction events.

### Concluding Remarks

The Arabidopsis mesophyll protoplast transient expression system has proven to be an informative system to characterize genes and pathways. However, the use of *Populus* mesophyll protoplast transient expression system is still in its infancy. The optimal plant growth conditions associated with highly efficient protoplast isolation, the optimal enzyme concentration and digestion time, the yield of protoplasts, the estimate of transfection efficiency, and the use of the system for studying the effect of exogenously-introduced genes had not been described previously. All of these parameters are essential for the efficient application of this system. We have reported these parameters here.

We established a protocol for the isolation of high yield, physiologically active and transfection-ready protoplasts from *Populus* leaves. We validated the suitability of this system for studying gene activity *in vivo* by showing the correct subcellular localization of introduced proteins, protein-protein interaction, gene activation, as well as the molecular responses of protoplasts to plant hormones. As a further test of the system, we observed a potentially conserved cellular energy signaling pathway in *Populus* protoplasts. The establishment of an efficient *Populus* protoplast isolation and transfection system provides a useful approach to characterize *Populus* genes and pathways *in vivo*.

Because protoplasts were derived from single cell type and deprived of cell wall, hypothesis and conclusions generated from transient expression study with this system should be cautiously analyzed and supplemented with whole plant studies. Furthermore, the development of protein extraction protocol for *Populus* protoplasts would help make the *Populus* protoplasts a more robust and reliable system for studying protein-protein interactions and their involvement in signaling events. Because *Populus* is a promising feedstock for lignocellulosic biofuel production, it is worthwhile to further investigate whether one could draw reliable conclusions from observations in leaf mesophyll protoplasts for lignin-producing tissue. We have showed that *Populus* mesophyll protoplasts responded to exogenous plant hormone treatments in a manner similar to intact leaves (Figure 4). Earlier studies in *Zinnia elegan* showed that protoplasts isolated from mesophyll can be induced to mature tracheary elements [39], [40], the units of xylem featured with various patterns of secondary cell wall deposition. In addition, ectopic expression of SND1, a transcription factor regulating the expression of secondary cell wall biosynthesis, induced ectopic deposition of secondary cell wall in mesophyll cells in *Arabidopsis* [41]. Taken together, these studies suggested that *Populus* mesophyll protoplasts have the potential to be used for dissecting the functional aspects of secondary cell biosynthesis and deposition. This represents a promising area for future investigation.

## Materials and Methods

### Plant Growth Conditions

Experiments were performed with micropropagated *Populus* clone 717-1B4 (female, *Populus tremula* x alba). Fresh, young shoots from plants grown in a greenhouse were used as starting materials for plant propagation in media after surface-sterilization [5 min in 1% (v/v) Tween-20 solution, 1 min in 70% (v/v) ethanol, 15 min in 10% (v/v) bleach solution and then triple rinsed for 5 min in sterile water]. The plants were maintained in Magenta boxes (GA-7) on 100 ml of MS medium with micro and macronutrients, vitamins, and glycine (Murashige and Skoog, Sigma-Aldrich) supplemented with 1% (w/v) sucrose, 0.5 g MES (2-(N-Morpholino) ethanesulfonic acid sodium salt) hydrate (Sigma-Aldrich) and solidified with 0.8% (w/v) agar, and pH adjusted to 5.7. The plants were cultivated in a tissue culture growth room: 25°C with a 24-h photoperiod. The light is provided by fluorescence tubes (cool white, 95 W, F96T12/CW/HO/SS, Sylvania) at an intensity of 70 µmol m^−2^ s^−1^ and plants were multiplied every 6 to 8 weeks [23].

### Protoplast Isolation

Healthy, fully expanded *Populus* leaves were cut into 1–2 mm fine strips and digested in an enzyme solution [0.4 M mannitol, 20 mM KCl, 20 mM MES-KOH (pH 5.7), 3% (w/v) cellulase R10 (Yakult Pharmaceutical Ind. Co., Ltd., Japan), 0.8% (w/v) macerozyme R10 (Yakult Pharmaceutical Ind. Co., Ltd., Japan), 10 mM CaCl_2_, 5 mM β-mercaptoethanol and 0.1% (w/v) bovine serum albumin (BSA)] in the dark for 5 h with gentle shaking on a Boeker Rocker II (Boeker Inc.). The protoplasts were harvested by filtering through a 70 µm pore nylon cloth (Carolina Biological Supply Company) and then suspended in a transfection buffer (0.4 M mannitol, 15 mM MgCl_2_, 4 mM MES-KOH (pH 5.7)) to a concentration of 1×10^6^ protoplasts ml^−1^.

### Protoplast Transfection

For each transfection assay used for qRT-PCR analysis, 50 µg plasmid [1 µg µl^−1^, prepared with Qiagen Midi Prep Kit (Qiagen Inc.)] was added to 500 µl of protoplast suspension, to which 550 µl PEG solution [40% (w/v) polyethylene glycerol (Sigma-Aldrich, MW 4000), 0.2 M mannitol, 0.1 M CaCl_2_] was added. For transfection assays used for GUS reporter analysis, 10 µg effector and 10 µg reporter plasmids were transfected with 100 µl protoplast, to which 120 µl PEG solution was added. The mixture was incubated at room temperature for 5 min before 2.2 ml of W5 solution [154 mM NaCl, 125 mM CaCl_2_, 5 mM KCl, 2 mM MES-KOH (pH 5.7)] were added to stop the reaction. The protoplasts were collected by centrifugation at 1,200 rpm (331 g) for 2 min on a swing-out rotor and then suspended overnight in 5 ml of incubation buffer [0.5 mM mannitol, 4 mM MES-KOH (pH 5.7), 20 mM KCl] in a 100 mm^2^ petri-dish.

### Imaging Methods

A set of vectors that transiently-expressed different organelle markers fused with mCherry [24] and an ubiquitously-expressed RACK1-YFP [25] were used for PEG-mediated transfection in *Populus* protoplasts. Images were captured using a Zeiss LSM 710 confocal laser scanning microscope equipped with a PlanAcromat 40x/1.4NA oil objective. Spectral imaging and linear unmixing were used to separate chloroplast autofluorescence from the mCherry-specific signal. Briefly, the cells were scanned with a 561 nm laser line and lambda-stack images were collected at a spectral resolution of 9.7 nm from 569–729 nm. Optical sections were collected at a z-scaling of 1 micron. After image acquisition, each set of lambda 3D confocal z-stacks was linearly unmixed with Zen 2009 software (Zeiss). The spectra (emission fingerprints) from unlabeled chloroplasts and the mCherry fusion proteins were unique in shape and were used to generate a 2-channel confocal stack representing chloroplasts (shown in red) and mCherry fusion protein (shown in green). Unmixed confocal stacks are displayed as maximum intensity projections using Zen 2009 software.

### Other Methods

Total RNA was extracted from 5×10^5^ protoplasts for each sample using Trizol® (Invitrogen Inc.). Two-hundred-fifty microliters of Trizol® was used for each RNA extraction, and 100 µg Ambion® Glycogen was added in the RNA precipitation step as carrier. Five hundred nanograms of total RNA was used for reverse transcription using RevertAid™ Reverse Transcriptase (Fermentas Inc.) and oligo dT_16_ was used as primer. The real-time PCR primers were designed using the NCBI Primer-BLAST tool [42]. The specificity of each pair of primers was determined by BLASTing the primers against Reference RNA sequence database for *Populus trichocarpa* (http://blast.ncbi.nlm.nih.gov/Blast.cgi). The real-time PCR reactions were conducted on a StepOne Plus™ Realtime PCR system (Applied Biosystems) with the iTaq^TM^SYBR® Green Super Mix with ROX (Bio-RAD Inc.). The expression of an ubiquitin gene *POPTR_01s44440* was used to standardize the expression of each gene. A 35S::GFP (ABRC stock #: CD3-911) construct was co-transfected for each samples to monitor the transfection efficiency for each assay. Only assays with >60% transfection efficiency were used for qRT-PCR analysis.

For KIN10-DIN6 study, AthKIN10 and PtrKIN10 was each cloned into a plant cell transient expression vector pE3225 [26]. The GFP sequence of pE3225 was removed and replaced with double HA (Human influenza hemagglutinin) tag. The genomic sequence 2 kb upstream of the start codon of *PtrDIN6-2* was cloned and fused to a *GUS* reporter gene by replacing the *UBQ10* promoter of the HBT95-pUBQ10-GUS construct [43]. For co-transfection assay, a 35S::LUC (Luciferase) was included in each transfection and the LUC activity was used to normalize GUS activity to account for the potential variations in the transfection efficiency [14]. The constructs used for BiFC were the same as those used previously [25].

For semi-quantitative RT-PCR, 1 µl cDNA for each sample was used. The PCR was conducted on a verti™ 96-well PCR machine (Applied Biosystems) for 30 cycles with the same primers used for qRT-PCR. All primers used in this study are listed in the Table S1.

For the detection of AthKIN10-HA, 2×10^5^ protoplasts were lysed in 2xSDS PAGE protein loading buffer (Bio-Rad) and the protein bands were separated in a 10% SDS-PAGE gel. The proteins were then transferred onto a PVDF membrane using a semi-dry transferring cassette (Bio-Rad). For western blotting, the membrane was blocked with TBS containing 0.05% Tween-20 and 2% non-fat dry milk for 1 h. Anti-HA-HRP (Roche Inc) was diluted to 1∶10,000 in TBS (25 mM Tris-HCl, 137 mM NaCl) and incubated with the blot for 1 h. The blot was washed three times (5 min each time) with TBS containing 0.05% Tween-20 and the signal was visualized by adding Pierce ECL western blotting substrate (Pierce Inc.) and exposure to X-ray film (Thermo Scientific).

For dark induction, the protoplasts were kept in the dark for 6 h whereas the control samples were kept in constant light with 70 µmol m^−2^ s^−1^. For hypoxia induction, protoplasts were settled at the bottom of a 2 ml Eppendorf tube filled with incubation buffer solution for 6 h while the control samples were kept in a 100 mm (diameter)×15 (height) mm petri-dish with 0.2 mm depth of solution. For hormone treatments, plant hormones with different concentration were added in the incubation buffer together with protoplasts or whole leaves and incubated for 3 h before they were harvested for qRT-PCR analysis. The method for GUS activity assay was essentially the same as described previously [14].

The *Populus* homologues were identified using the Phytozome web tool (http://www.phytozome.net) [44]. Briefly, the locus numbers of Arabidopsis genes were used to locate the Arabidopsis gene. The closest *Populus* homologs identified under the “homolog peptide” tool were chosen as candidate homologues.

All of the primer sequences used in this paper are listed in the Table S1.

## Supporting Information

Figure S1 Representative images of Evans Blue staining of *Populus* protoplasts.
(TIF)

Figure S2 The expression of three *Populus KIN10* homologues determined by the eFP tool (bar.toronto.ca).
(TIF)

Table S1 List of primers used in this study.
(XLSX)
