# Supplementary figures and images for: Highly Efficient Isolation of Populus Mesophyll Protoplasts and Its Application in Transient Expression Assays
Source: PLoS One. 2012 Sep 13;7(9):e44908. doi: 10.1371/journal.pone.0044908 (PMC3441479; doi:10.1371/journal.pone.0044908)

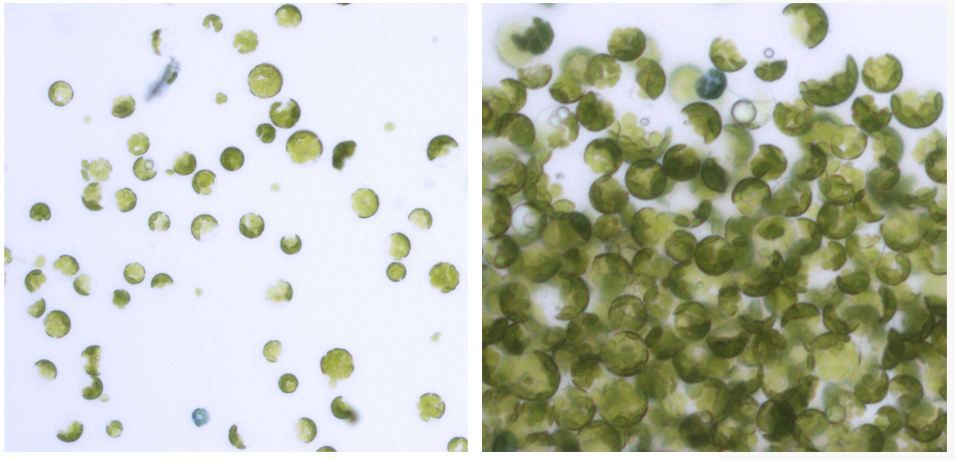

Supplement: Figure S1 — Representative images of Evans Blue staining of Populus protoplasts. (TIF) [file pone.0044908.s001.tif]

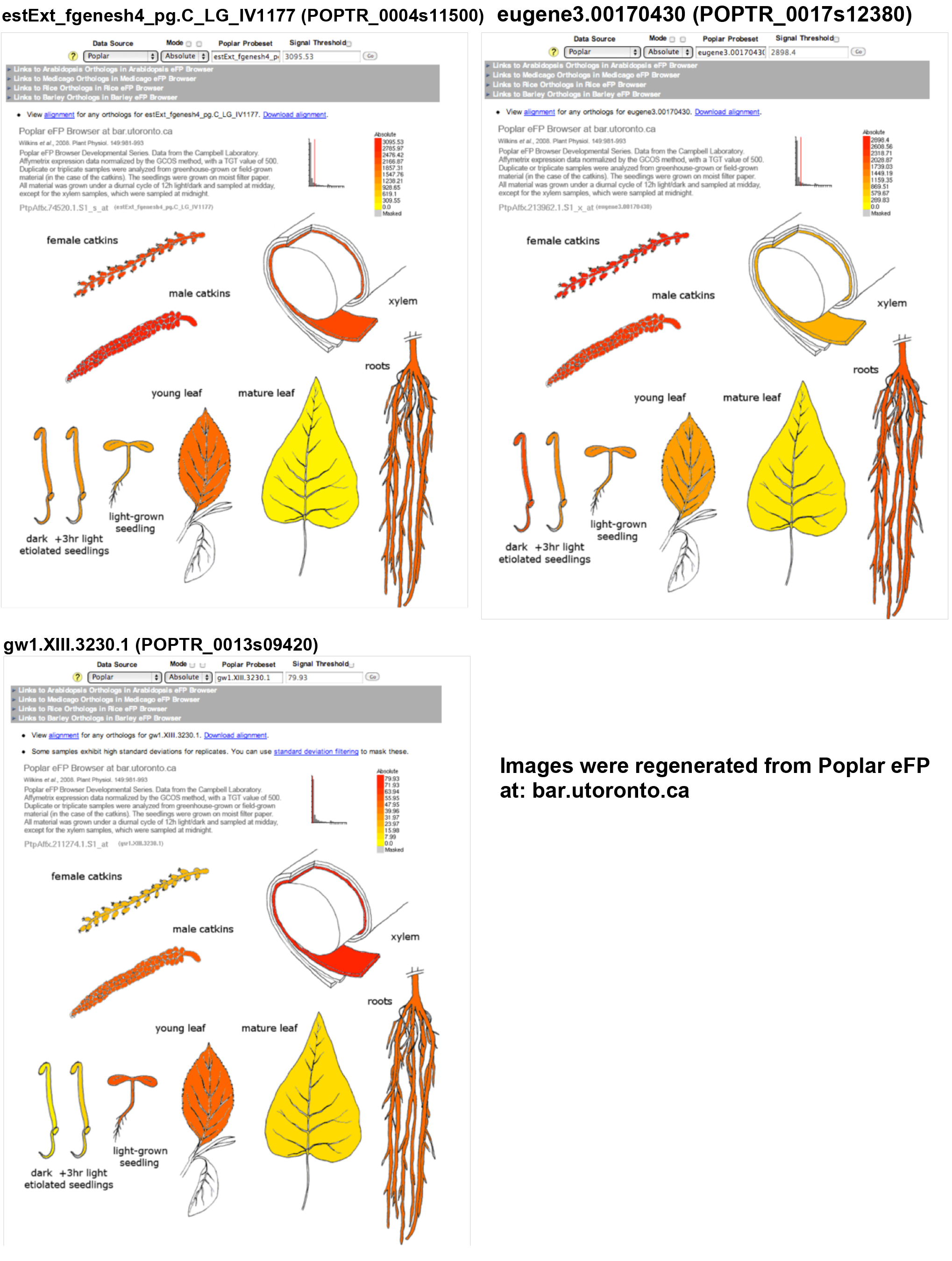

Supplement: Figure S2 — The expression of three Populus KIN10 homologues determined by the eFP tool (bar.toronto.ca). (TIF) [file pone.0044908.s002.tif]
